# Reverse Anti-solvent Crystallization Process for the Facile Synthesis of Zinc Tetra(4-pyridyl)porphyrin Single Crystalline Cubes

**DOI:** 10.1038/s41598-017-02718-9

**Published:** 2017-05-31

**Authors:** Yohwan Park, Misun Hong, Jin Young Koo, Minkyung Lee, Jinho Lee, Dae Jun Moon, So Hyeong Sohn, Taiha Joo, Woo Taik Lim, Hyunseob Lim, Hee Cheul Choi

**Affiliations:** 1 Center for Artificial Low Dimensional Electronic Systems, Institute for Basic Science (IBS), Pohang, 37673 Republic of Korea; 2 Department of Chemistry, Pohang University of Science and Technology (POSTECH), Pohang, 37673 Republic of Korea; 3 Department of Chemistry, Korea Advanced Institute of Science and Technology, 291, Daehak-ro, Yuseong-gu, Daejeon 34141 Korea; 4 Department of Applied Chemistry, Andong National University, Andong, 36729 Republic of Korea; 5 Department of Chemistry, Chonnam National University, Gwangju, 61186 Republic of Korea

## Abstract

Synthesis of morphologically well-defined crystals of metalloporphyrin by direct crystallization based on conventional anti-solvent crystallization method without using any additives has been rarely reported. Herein, we demonstrate an unconventional and additive-free synthetic method named reverse anti-solvent crystallization method to achieve well-defined zinc-porphyrin cube crystals by reversing the order of the addition of solvents. The extended first solvation shell effect mechanism is therefore suggested to support the synthetic process by providing a novel kinetic route for reaching the local supersaturation environment depending on the order of addition of solvents, which turned out to be critical to achieve clean cube morphology of the crystal. We believe that our work not only extends fundamental knowledge about the kinetic process in binary solvent systems, but also enables great opportunities for shape-directing crystallization of various organic and organometallic compounds.

## Introduction

**Figure 1:**
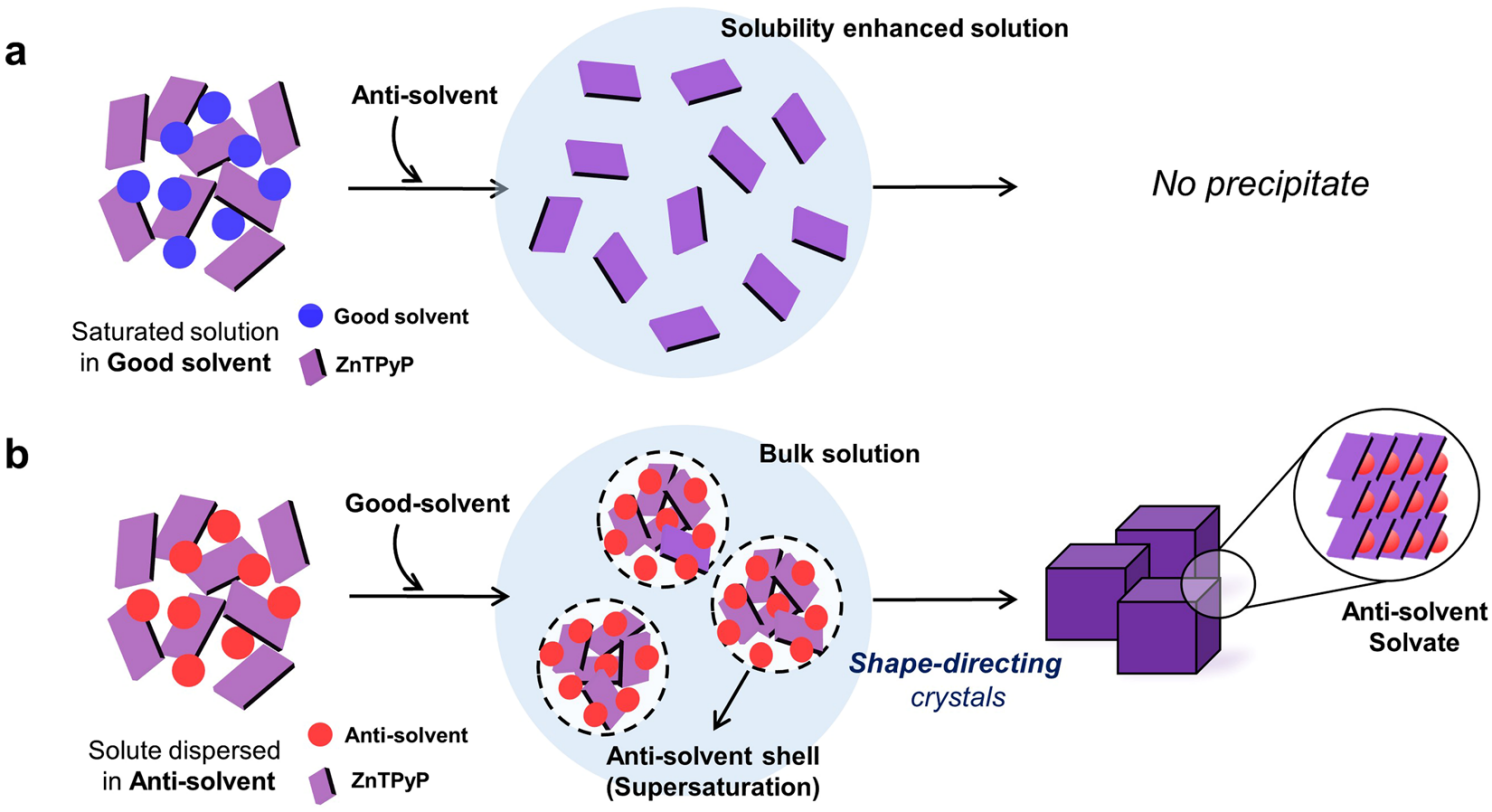
Scheme of *c*-ASC and *r*-ASC methods. (**a**) Conventional anti-solvent crystallization (*c*-ASC) method and (**b**) reverse anti-solvent crystallization (*r*-ASC) method, which reverse the solvent-addition order compared with the *c*-ASC method.

The morphological shape of micro- or nano-crystals highly influences their physical, biological, and chemical properties^1–5^. Therefore, development of the synthesis of organic and organometallic crystals in well-defined morphologies has been considered as an important research subject. During the last decade, our group has been investigating synthetic methods for various organic- and metal-containing highly conjugated molecular crystals^6–16^. One of the representative tetrapyrrole molecules, porphyrin derivatives, constitute attractive building blocks for organic micro- or nano-structures due to their distinct electronic, optical, and catalytic properties^17–20^. In 2009, we synthesized metal-free porphyrin rectangular tube crystals by the physical vapor transport (PVT) method^14^, and confirmed that the crystals are constructed by several intermolecular interactions, such as hydrogen bonding, hydrogen-π, and π-π intermolecular interactions. However, the shape-specific synthesis of metal-containing porphyrin (metalloporphyrin) crystals by the PVT method has not been successful mainly due to difficulties in providing a reaction environment for optimized kinetic control. This is a result of the nature the PVT process, which is not ideal to provide a homogeneous environment for molecules to make such intermolecular interactions fully applied to allow several key factors, including freedom of molecules, to form the appropriate dynamic motions necessary for the required intermolecular interactions to be effective within the time scale of the intermolecular interactions for the formation of crystal nucleates. Compared with metal-free porphyrin, for example, metalloporphyrin, might be needed to make metal-ligand coordination interactions to form morphologically well-defined crystals. However, as previously mentioned, it is difficult for the PVT method to provide a reaction environment that enables all of the feasible intermolecular interactions. On the other hand, a solution phase reaction would be an ideal approach to overcome this limitation. In fact, several studies have reported that the shape-specific synthesis of metalloporphyrin in the solution phase can be accomplished by adding additives, such as acid, surfactant, etc.^21–27^, which facilitate kinetic control by accelerating intermolecular interactions or by minimizing the surface energy of certain crystal planes. It is clear, however, that the addition of extra chemicals should be minimized in order to maintain the original properties of the target crystals. From this perspective, our synthetic method is ideal and superior. We have synthesized cube-shaped crystals of zinc 5,10,15,20-tetra(4-pyridyl)-21H,23H-porphyrin (ZnTPyP) for the first time without any additives, but simply using only solvent-solvent and solvent-solute interactions in binary solvent systems based on the concept of the *c*-ASC method, which is one of the most widely used crystallization methods. When the *c*-ASC method was utilized, however, ZnTPyP crystals (or even any precipitates) were not formed mainly due to the failure of achieving the supersaturation environment due to the unexpected increase in the solubility of the solute in the toluene/IPA binary solvent system. Therefore, we slightly modified the *c*-ASC method, as it provides a local supersaturation environment well, and resolved the problem. Herein, we demonstrate this novel and facile synthetic method (*r*-ASC method, *vide infra*), which first roughly disperses ZnTPyP powder precursor molecules into the anti-solvent, and then adds the good solvent to form a local supersaturation environment (Fig. 1). To explain the precise mechanism, we first introduce the solvation shell effect (FSSE) mechanism^28–30^, which successfully explains the dissolution of ionic compounds in polar solvents through the effect of hydration or solvation of ionic solute with water or polar solvents by making its first solvent shell around the solutes. However, the conventional FSSE mechanism possesses a limitation to explain *r*-ASC and *m*-ASC methods that target neutral solutes and non-polar anti-solvents. Herein, in advance of the conventional FSSE mechanism, we suggest an extended FSSE mechanism that is suitable to explain the critical role of the type of solvation solvent regardless of the species of solvents (e.g., polar and non-polar solvents) that can be simply controlled by changing the solvent-addition order. This suggests a new kinetic route for reaching the local supersaturation environment and enables shape-specific crystal synthesis.

## Results and Discussion

### Design of the new facile method to overcome limitation of anti-solvent crystallization method

To prove this, we first attempted the *c*-ASC method for ZnTPyP powder solutes with isopropyl alcohol (IPA) as a good solvent, and toluene as an anti-solvent. For this, an excess amount of as-received ZnTPyP powder (Sigma-Aldrich, 90%) was added first in IPA (Sigma-Aldrich, ≥99.9%) to form a ZnTPyP-IPA supersaturated solution. The ZnTPyP-IPA supersaturated solution was then filtered to prepare a saturated ZnTPyP-IPA solution. Then, the anti-solvent (toluene) was slowly added into the saturated-ZnTPyP-IPA solution. More details about the experimental details are described in Method section. Interestingly, a quite unexpected result was found, in which no precipitation was observed during this process (Fig. S1). This is because the addition of an anti-solvent into a solution saturated with solutes in a good solvent generally induces the decrease of the total solubility of the solute in a binary solvent system, which readily results in prompt precipitation. However, an increase of total solubility of ZnTPyP was rather observed from 0.1330 mM in the saturated IPA solution to 0.3478 mM in the IPA/toluene binary solvent system (Fig. S2). Such phenomenon has been previously studied and explained by Hansen solubility parameters that consider dispersive, polar, and hydrogen bonding solubility parameters for several solute and solvent mixtures^31^. Therefore, the solubility enhancement demonstrates that the c-ASC method is not appropriate for the crystallization of ZnTPyP when IPA and toluene are used for good and anti-solvent, respectively. In order to overcome this problem in the *c*-ASC method, we attempted two modified reactions: i) Toluene was added into the supersaturated ZnTPyP-IPA solution without filtration, under mild hand-shaking. No crystal with specific morphology was still obtained, but irregular precipitates were achieved (this method is termed the ‘*modified-*ASC (*m*-ASC) method’). ii) By reversing the solvent-addition order (termed the ‘*reverse* ASC (*r*-ASC) method’), i.e., we dispersed the same amount of ZnTPyP precursor in the toluene anti-solvent first, and then IPA was added to the solution under an identical reaction condition. More details about the experimental details are described in Method section. Although both processes produced precipitates in the binary solvent system, ZnTPyP cube crystals were obtained only from the latter case.

### Morphology and structure analysis of crystals synthesized by *r*-ASC and *m*-ASC methods

**Figure 2:**
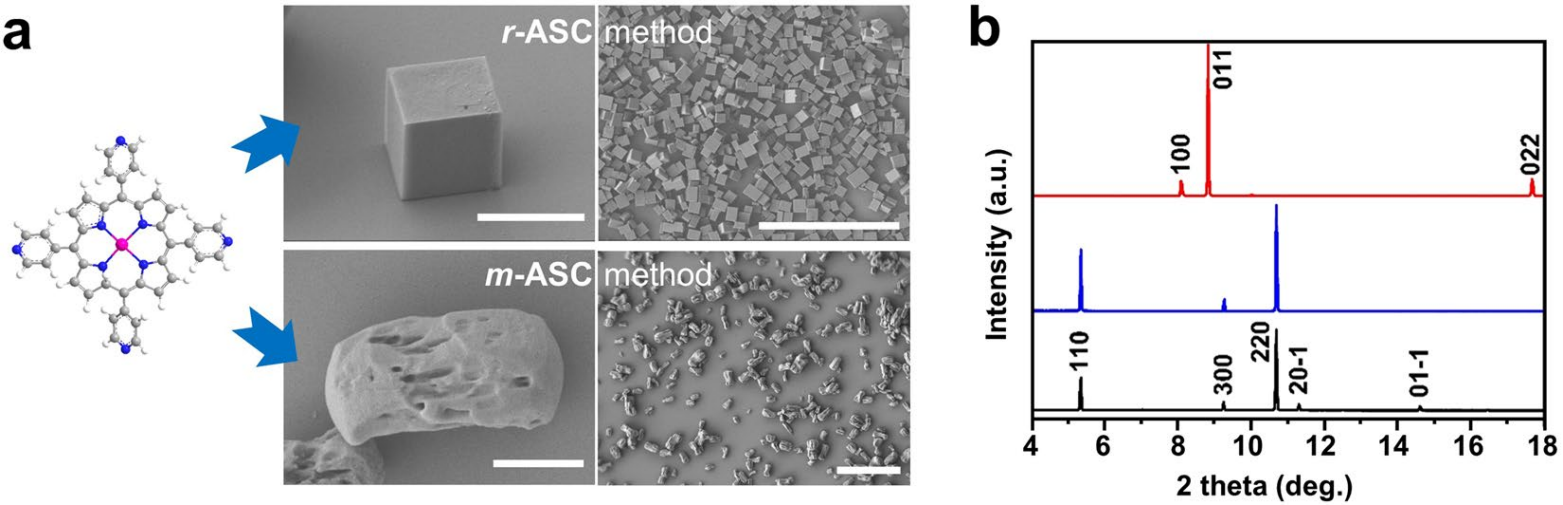
SEM images of the crystals synthesized by *r*-ASC and *m*-ASC method and their powder XRD patterns. **(a**) High magnification (left column, scale bar = 5 μm) and low magnification SEM images (right column, scale bar = 50 μm) of ZnTPyP cubes and ZnTPyP precipitates grown by *r*-ASC and *m*-ASC method, respectively. (**b**) XRD patterns of commercial ZnTPyP powder (black), irregular precipitates obtained by the *m*-ASC method (blue), and cube-shaped crystals obtained by the *r*-ASC method (red). All of the spectra were converted to the wavelength of the Cu Kα1 line (λ = 1.54057 Å).

**Figure 3:**
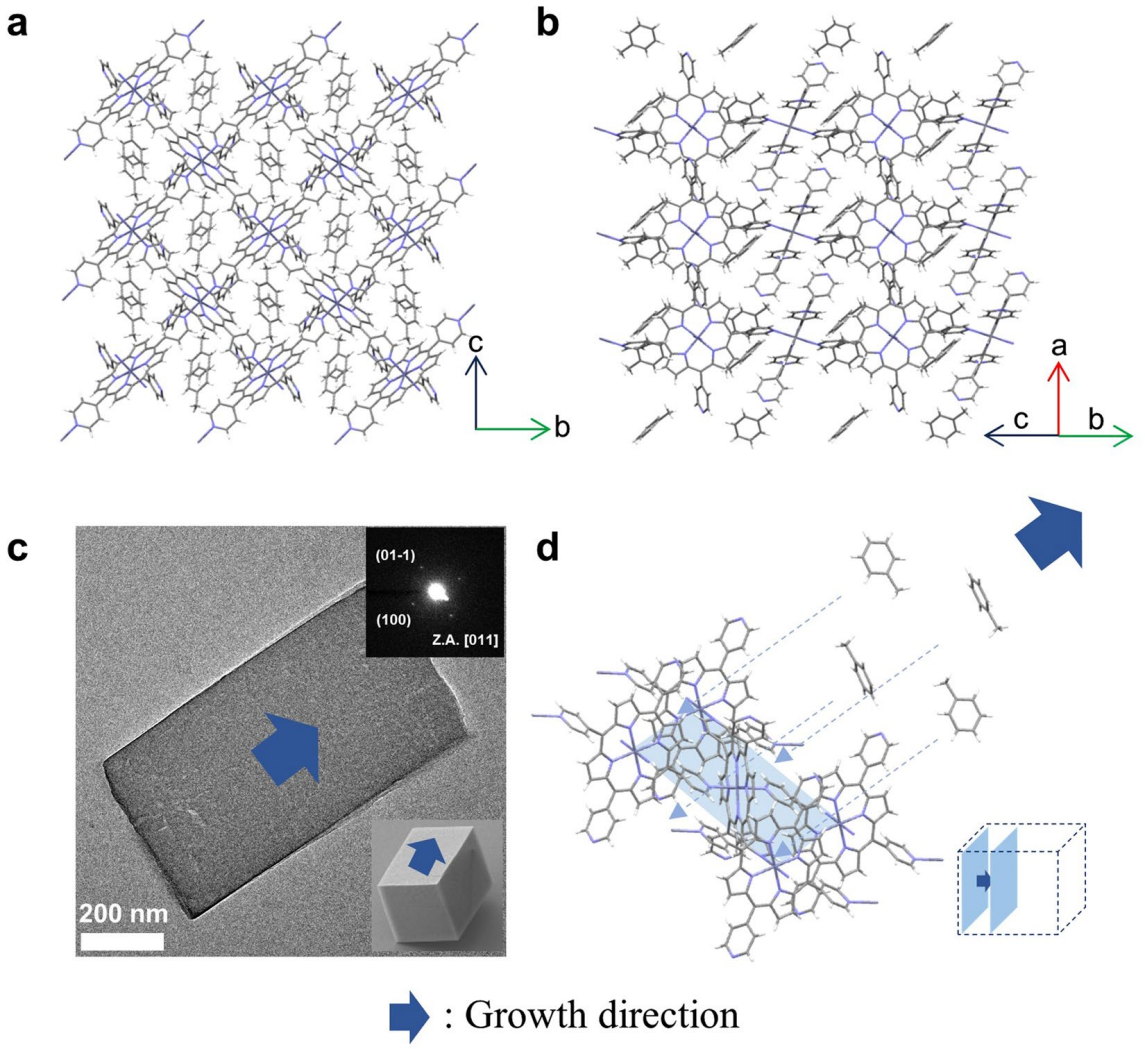
Single crystal structure and TEM analysis of ZnTPyP cube crystal. **(a**,**b**) Single crystal space-filling representations of (100) and (011) plane of cube crystal, respectively. (100) plane shows 2D coordination polymer of ZnTPyP including zinc metal-N axial coordination bonding, and (011) plane shows intercalation of toluene into the 2D coordination polymer. (**c**) TEM image and (inset) selected area electron diffraction (SAED) pattern of square pillar shaped-ZnTPyP corresponding to the TEM image. The arrow represents the growth direction of the ZnTPyP cube, and the zone axis is identified as (011). (**d**) Representation of growth direction introduced by toluene solvent molecule into 2D ZnTPyP coordination polymer.

The morphology and size of ZnTPyP precipitates were characterized by scanning electron microscopy (SEM). The ZnTPyP precipitates having non-uniform morphology were obtained by the *m*-ASC method (Fig. 2a) and we confirm that the ZnTPyP precipitates formed by the *m*-ASC method are similar with the initial ZnTPyP powder in Fig. S10, which indicates that no recrystallization occurs by the *m*-ASC method different from *r*-ASC method. In contrast, the well-defined cube shape (or square pillar shape) crystals with homogeneous size of 3–5 μm were obtained in the precipitates by the *r*-ASC method (ZnTPyP cube), and the cubes exhibited highly smooth surfaces. Their crystal structures were investigated by powder X-ray diffraction (*p*XRD) measurements. The *p*XRD patterns in Fig. 2b reveal that ZnTPyP precipitates (blue) by the *m*-ASC method have the same crystal structure as that of as-received ZnTPyP precursor powder (black), while ZnTPyP cubes have a totally different crystal structure (red). To elucidate the detailed crystal structure and intra- and inter-molecular interactions, ZnTPyP cube was further investigated by single-crystal XRD (*s*XRD) measurement using a synchrotron X-ray beam source. A ZnTPyP cube crystal has a monoclinic unit cell with a = 10.917(2) Å, b = 13.850(3) Å, c = 14.208(3) Å, α = 90°, β = 91.00(3)°, γ = 90° (Fig. 3a and b). In addition, its space group is assigned to be P2_1_/c, which is determined by the program XPREP^32^. Based on the *s*XRD information, lattice planes for *p*XRD peaks in Fig. 2b (red) are assigned to originate from (100), (011) and (022) planes, respectively. In Fig. 3a and b, ZnTPyP molecules in a ZnTPyP cube form a 2D coordination polymer by zinc metal-N axial coordination bonding interaction between (100) and (011) planes. These 2D coordination polymer layers are stacked along the a-axis [100] direction, which indicates the intercalation of toluene molecules as a guest molecule. The solvated toluene molecules could be accommodated into ZnTPyP 2D coordination polymers for the formation of well-defined cube crystals. This result agrees well with the previously reported crystal structure of 1,2-dichlorobenzene solvated ZnTPyP crystal^33^. Transmission electron microscopy (TEM) combined with selected area electron diffraction (SAED) pattern further assigns the lattice planes for three facets of the ZnTPyP cube. Figure 3c shows the TEM image of the square pillar shape ZnTPyP crystal, which was chosen because it is more appropriate for TEM study since the information of anisotropic facets can be additionally determined. Each spot in the rhombic SAED pattern in the inset of Fig. 3c corresponds to lattice spacing values of (100) and (01–1) planes, estimated from *s*XRD, and thus its zone axis can be identified as the [011] direction. Consequently, one square face is a (100) surface, and the other rectangular two facets are (011) and (01–1) surfaces. Structural analysis indicates that toluene guides to grow ZnTPyP molecules from 2D coordination polymer into a 3D cube shaped crystal, by assembly through zinc-nitrogen six-coordination (Fig. 3d). In contrast, the *p*XRD pattern of the ZnTPyP precipitates obtained by the *m*-ASC method is unchanged compared with the ZnTPyP precursor, which matches well with the *p*XRD pattern of the previously reported pattern, known as a trigonal crystal with R3 space group^34^. Lattice planes for *p*XRD peaks in Fig. 2b (blue) are assigned as (110), (300), and (220) planes. In addition, the structure was constructed by forming zinc-nitrogen six-coordination stabilized by hydrogen bonding with H_2_O or methanol as *s*XRD information of the reference (See Supplementary Information for detailed crystal structures, Fig. S3). Judging by the correlation between the crystal structure and species of solvent, solvents containing hydrogen bonding that enable the site could not cause the recrystallizing of ZnTPyP molecules due to their already being structurally stable about these solvents. Accordingly, the IPA solvation shell could not act as driving force for insertion of the IPA-like toluene solvation shell during the *m*-ASC method process.

### Extended first solvation shell crystallization mechanism

The solute-solvent interaction has been studied to explain various phenomena in binary solvent systems. Especially, preferential solvation has been described importantly in terms of solute-solvent and solvent-solvent interactions, as well as solvent-exchange equilibria^35^. The solvent-exchange equilibria show an exchange model between the local solvation environment and the bulk solution environment around the solute, but the control of the dominant solvation environment and prediction of solvated solvent have been rarely studied. In order to prove the obviously different crystallization behaviors depending on the solvent-addition order, which influences the critical crystallization environment, i.e., different first solvation shell, we suggest a new kinetic crystallization mechanism, the extended FSSE mechanism, which is based on the preferential solvation theory^36^. The conventional FSSE mechanism that explains the effect of polar solvents for the local environment of ionic compounds has a limitation to describe *r*-ASC and *m*-ASC methods, which target neutral solutes and solvents, regardless of their species. Accordingly, the extended FSSE mechanism indicates that the solvation shell formed by the first-added solvent, regardless of the species of solvent, highly influences the crystallization of neutral solute by control of the local supersaturating environment. When toluene anti-solvent is added first to ZnTPyP, in the *r*-ASC method, it could be dispersed only roughly, and the toluene molecules make a solvation-shell rapidly with preferential binding, such as the van der Waals (*vdW*) interaction and π-π interaction. As soon as IPA good solvent is added, the toluene solvation-shell inhibits interaction between IPA and ZnTPyP, and the shell is localized in the solution. Then, the shell forms the local supersaturation environment and promotes the crystallization of solutes into specific solvated crystals. Contrary to the *r*-ASC method, in the *m*-ASC method, solvation-shell is formed by the first-added solvent, IPA, caused by interaction such as ligation, hydrogen bonding, and dipole-dipole interaction. The highly interacting IPA with ZnTPyP interrupts the solvation of toluene molecules into precipitation, which induces poor crystallization. The same phenomena were observed when using the geometrically similar anti-solvent species, chlorobenzene, 1,2-dichlorobenzene, and *o*-xylene. As shown in Fig. S9, although different anti-solvents were used, the same cube (or square pillar) shape crystals with the same crystal structure were obtained. This means that the geometrically similar anti-solvents have similar intermolecular interactions with ZnTPyP by forming their solvation-shell, and shape-directing synthesis occurs during the *r*-ASC method.

**Figure 4:**
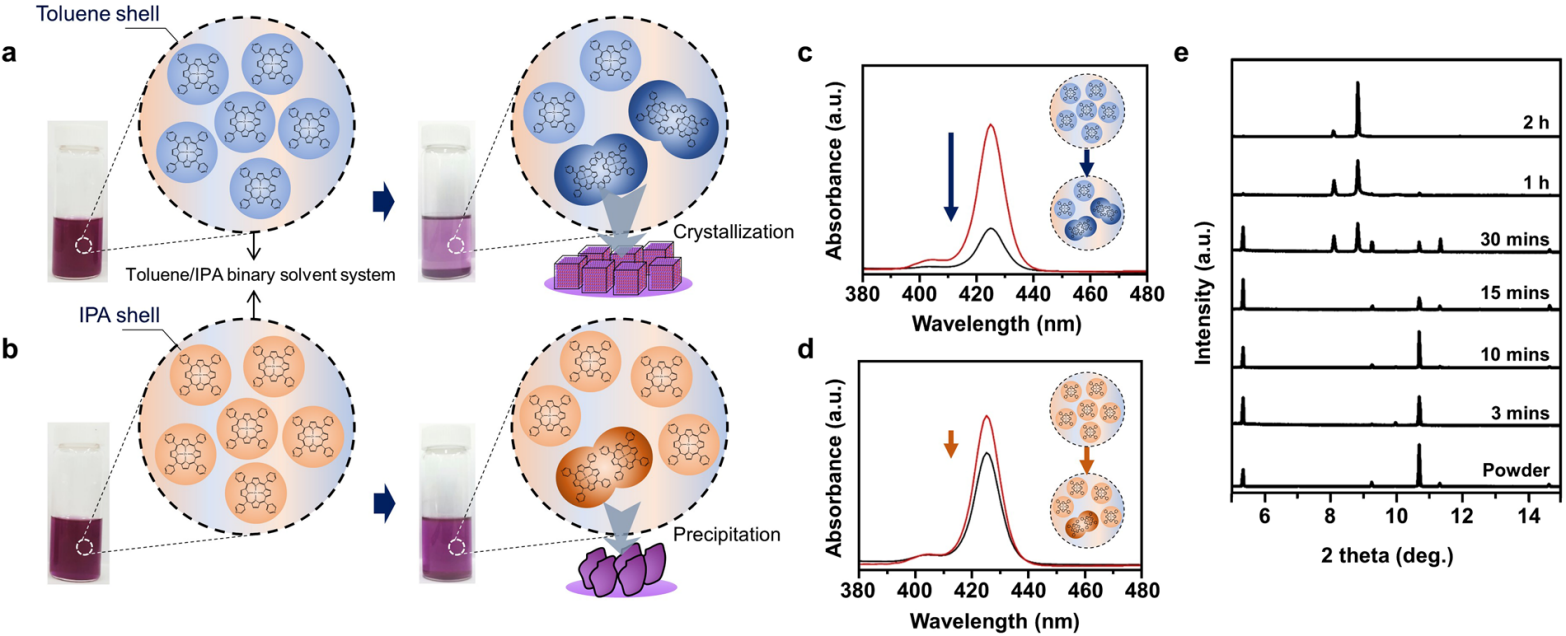
Extended first solvation shell mechanism and its indirect evidence *via* UV/VIS spectra and time-dependent XRD patterns. (**a**) Schemes and photographs of the *r*-ASC method, including toluene solvent-shelled ZnTPyP that promotes crystallization to cube crystal, and excess powder is dissolved to maintain supersaturated solution. (**b**) Schemes and photographs of the *m*-ASC method, including IPA solvent-shelled ZnTPyP that protects ZnTPyP to crystallize to ZnTPyP cube, and excess powder is precipitated. (**c**,**d**) UV/VIS spectra of as-received solution by the *r*-ASC method and the *m*-ASC method (red), respectively, and after 1 day (black). (**e**) XRD patterns of crystals grown by the *r*-ASC method depending on reaction time.

To demonstrate the extended FSSE during either *m*-ASC or *r*-ASC processes, the total solubility of ZnTPyP in the binary solvent system has been monitored according to process time by UV-VIS spectroscopy, depending on the solvent-addition order. UV-VIS absorption spectra and optical photograph in Fig. 4 revealed the different concentration of ZnTPyP in the solutions with different solvent-addition order, 2 h after adding the second-solvent despite the identical amount of ZnTPyP, toluene, and IPA. To calculate accurate concentration change upon *r*-ASC and *m*-ACS processes, final solutions having excess powder were filtered and measured UV-VIS absorption from diluted solutions as Fig. 4c and d. The absorption peak intensities of the two solutions were initially almost identical (0.3478 mM), but the concentration changes were obviously different as 0.2148 and 0.2620 mM after 1 day upon *r*-ASC and *m*-ASC, respectively. The solution into which toluene was added first exhibits a considerable change of concentration (Fig. 4c), because the toluene solvent-shelled ZnTPyP forms first, and this shell induces a local supersaturation environment, and thus the continuous growth of ZnTPyP cubes in the binary solvent system. On the other hand, the solution into which IPA was added first shows a slight change of concentration (Fig. 4d) because the IPA solvent-shelled ZnTPyP forms first, and thus the shell prevents crystallization from solvation of toluene. The results demonstrate that the first-added solvent determines the type of solvation-shell around ZnTPyP molecules, which highly influences the crystallization of ZnTPyP. The change of crystal structure in the precipitation as a function of growth time was also investigated by XRD. During the *r*-ASC process, in Fig. 4e, only ZnTPyP powder XRD pattern was observed during the first 15 min of the reaction, and ZnTPyP cube and powder pattern started to appear simultaneously after 30 min of the reaction. Finally, only the ZnTPyP cube pattern was found after 2 h of the reaction. This means that, after 30 min of the reaction, excess ZnTPyP powder started to be dissolved in the solution with the remaining toluene solvation-shell as the solvated solutes are crystallized out. In addition, these toluene solvent-shelled ZnTPyP molecules are crystallized into ZnTPyP cubes continuously until 2 h (See Supplementary Information for detailed SEM images of time-dependent experiment, Fig. S6). In contrast, the ZnTPyP powder pattern was invariably observed during the whole reaction process when the *m*-ASC method was used (Fig. S7). All of the control experiments by *r*-ASC and *m*-ASC exhibited similar behaviors, which indicates that the *r*-ASC method and extended FSSE mechanism can be applied to various syntheses of organic and organometallic crystals.

### Photoluminescence properties

**Figure 5:**
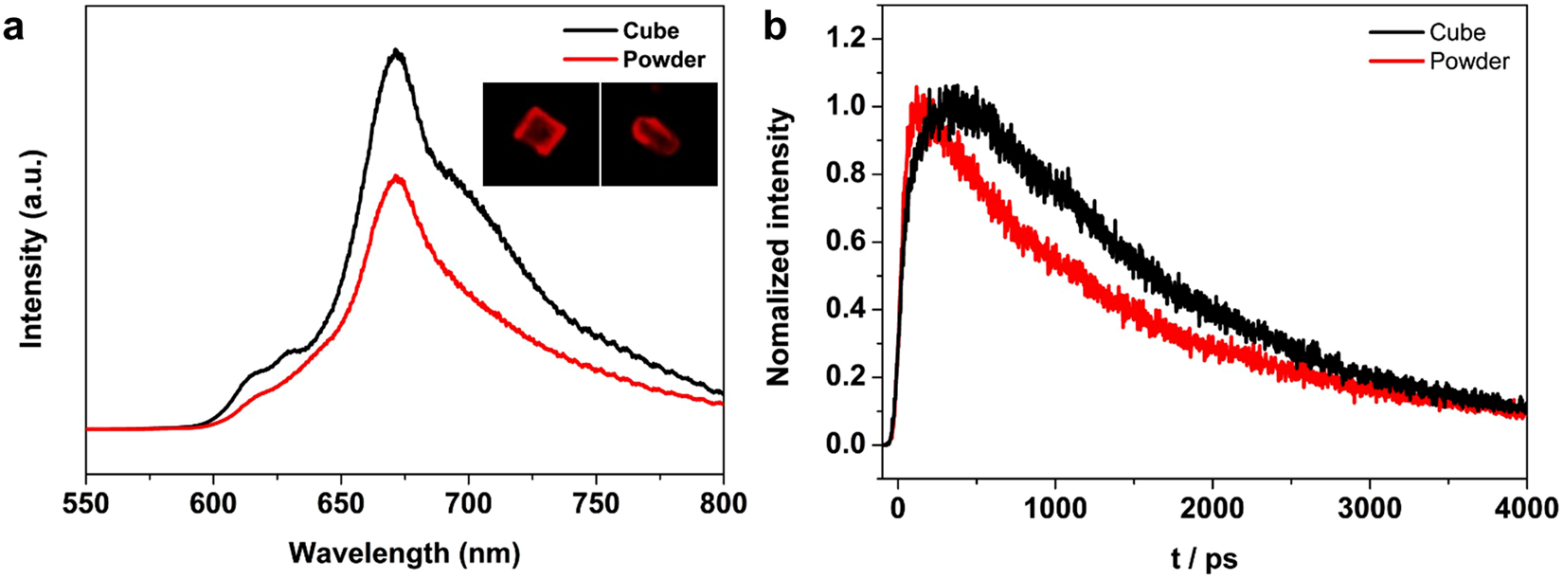
Photoluminescence properties of ZnTPyP powder and crystals. (**a**) Photoluminescence spectra of powder (red) and cube (black) crystal, respectively (in the inset, photoluminescence images of individual powder (left) and cube (right) crystal) (λ_laser_ = 338 nm and light source is a mercury lamp). (**b**) Time-resolved photoluminescence measurement of powder (red) and cube (black) crystal (detection λ_laser_ = 667 nm).

We have also investigated the optical properties resulting from the different crystal shapes. Steady-state photoluminescence (PL) images and PL spectra (Fig. 5a) were obtained from individual ZnTPyP cube and powder crystal, and the PL spectra were averaged from 10 samples of each crystal. It is found that the higher crystallinity of ZnTPyP results in higher PL intensity, which can be understood by crystallization-induced emission enhancement (CIEE)^12, 37^. The PL peak position is almost the same for the cube and powder, which implies that the solvated-toluene molecules in the ZnTPyP cube do not affect the PL property of ZnTPyP significantly. However, it is worth noting that the PL spectrum of the ZnTPyP cube exhibits two additional small shoulders at 629 and 763 nm. The visible absorption/emission features of porphyrins consist of Q_x_ and Q_y_ bands, and their activities depend critically on symmetry and conformation. The appearance of a band at 629 nm indicates that there is some change of the structure between the powder and cube forms. The triplet emission of porphyrin usually appears in the 720~800 nm region, and the 763 nm band can be assigned to the emission from a triplet. It is interesting to observe that the intersystem crossing rate is enhanced in the single crystal, although a detailed mechanism is not clear. The more detailed emission profile was investigated by time-resolved PL spectroscopy (TRPL). The TRPLs of ZnTPyP shown in Fig. 5b was obtained by the excitation of the Soret (S_2_) band in absorption spectrum (Fig. S4) by femtosecond pulses at 425 nm. The TRPL of ZnTPyP cube rises by a time constant of 210 ps (37%) and decays biexponentially by time constants of 1.3 ns (55%) and 2.7 ns (8%). In contrast, the TRPL of ZnTPyP powder rises much faster by a time constant of 34 ps (37%) and decays faster by two time constants of 860 ps (37%) and 2.3 ns (26%). The increase of the decay time constants of the ZnTPyP cube is consistent with the higher PL intensity of the ZnTPyP cube, as shown in Fig. 5a. In the TRPL of the Q_x_ band at 667 nm (Fig. 5b), the ZnTPyP cube exhibits a much slower rise (210 ps), compared to that of ZnTPyP powder (34 ps). The picosecond rise of the Q_x_ emission band has been observed previously, and was assigned to the vibrational relaxation within the Q_x_ state^38, 39^. The unexpectedly large decrease of the thermal dissipation rate through vibrational relaxation in the crystal phase may be caused by the reduction of the density of state (DOS) of solvent due to the smaller number of solvents. In addition, the reduction of the DOS of ZnTPyP may possibly be due to the more rigid structure in the crystal phase, which may also cause the reduced non-radiative relaxation rate to result in the longer lifetime.

## Conclusion

In summary, our work constitutes the first demonstration of the synthesis of well-defined cube-shaped ZnTPyP crystals. While the shape-directing synthesis of polyhedron metalloporphyrin crystal has been regarded to be difficult due to its direct-assembly or precipitation of target solute molecules using the conventional *c*-ASC method, we could overcome these limitations by controlling the solvation condition. By the *r*-ASC method, the local environment of ZnTPyP could be kinetically controlled to promote toluene molecules solvated around ZnTPyP. Systematic investigations revealed that the first-added toluene molecules could act as a key factor for the crystallization into cube-shaped morphology. In contrast, IPA molecules during the *m*-ASC method prevented crystallization from solvation of toluene. The detailed mechanism is newly suggested by the extended FSSE mechanism, which explains that controlling the first solvation shell by the solvent-addition order affects shape-directing synthesis remarkably. In addition, the improved optical property of the ZnTPyP cube was revealed, which is regarded as due to the crystallization-induced effect. We believe that this novel concept would not only extend fundamental knowledge of the kinetics in solute-solvent interactions, but also prompt new attempts at elucidating the crystallization route for various organic and organometallic compounds.

## Methods

### Synthesis

i) *c*-ASC method: *c*-ASC method: an excess amount of ZnTPyP powder (4 mg, Aldrich, 90%) was dissolved in 4 mL of IPA (Sigma-Aldrich, ≥ 99.9%) by ultrasonication (UCS-10, Lab companion) for 1 min, and ZnTPyP/IPA solution was filtered by a syringe filter having pore size of 0.02 μm. The final concentration is 0.1330 mM calculated by UV-VIS spectra (Fig. S2b and c) and 8 mL of toluene was added to ZnTPyP/IPA saturated solution, and the mixture was mixed by hand-shaking. The final mixture was kept at room temperature for 2 h.
ii) *m*-ASC method: 4 mg of ZnTPyP powder was dissolved in 4 mL of IPA with hand-shaking for 1 min, and 8 mL of toluene was added without filtration process (0.5387 mM). Then, the mixture exhibited a dark red color after mixing by hand-shaking for 1 min, and kept at room temperature for 2 h.
iii) *r*-ASC method: 4 mg of ZnTPyP powder was dispersed in 8 mL of toluene with hand-shaking for 1 min, and 4 mL of IPA was added slowly without a filtration process (0.5387 mM). The interface of IPA and ZnTPyP/toluene solution could be clearly observed, and a dark red color was observed when the mixture was mixed by hand-shaking for 1 min. Then, the final mixture was kept at room temperature for 2 h.

### Time-dependent XRD measurement

To monitor the degree of crystallization as a function of time upon *r*-ASC and *m*-ASC method, each solution was newly prepared according to time in same batch and extract, drop and dry 12 μL the solution by pipette on Si substrate to measure *p*XRD. 4 mg of ZnTPyP was dispersed in 8 mL of toluene and added 4 mL of IPA slowly along the vial wall and only the solvent adding order was reversed in *m*-ASC method.

### Characterization

All of the SEM images were obtained using a JEOL, JSM-7410F instrument, and TEM images and electron diffraction patterns were obtained using a JEOL JEM-2100F (with Cs Corrector on STEM) instrument. To investigate the detailed crystal structure, powder XRD and single crystal XRD data were collected by using a synchrotron (Pohang Accelerator Laboratory (PAL), 5D and 2D beamline, λ = 1.23954 Å). All obtained X-ray diffraction data were converted to Cu K_α_ (λ = 1.54057 Å) radiation.

For single crystal XRD, the temperature was maintained at 100(1) K by a flow of cold nitrogen gas. Preliminary cell constants and an orientation matrix were determined from 72 sets of frames collected at scan intervals of 5° with an exposure time of 1 s per frame. The basic scale file was prepared using the HKL3000sm program. The reflections were successfully indexed by the automated indexing routine of the DENZO program^40^. The diffraction data were harvested by collecting 72 sets of frames with 5° scans with an exposure time of 1 s per frame. These highly redundant data sets were corrected for Lorentz and polarization effects, and a very small correction for crystal decay was applied.

UV/VIS spectra were obtained using a spectrometer (Shimadzu 2600).

Steady-state photoluminescence spectroscopy and imaging of powder and cube crystal were obtained using a fluorescence microscope (Olympus microscope) equipped with a fluorescence filter (λ_ex_ = 330–380 nm, λ_em_ = 420 nm long pass filter). The picosecond time-resolved photoluminescence was measured by the time-correlated single photon counting (TCSPC) technique. Fundamental femtosecond pulse came from home-built cavity dumped Ti:sapphire laser, which was pumped by second harmonics of Nd:YVO_4_ continuous wave laser (Verdi, Coherent). Fundamental pulse centered at 850 nm was frequency-doubled at 500 μm β-barium borate crystal to excite porphyrin samples with a repetition rate of 355 kHz. The fluorescence was dispersed by a monochromator (SP-300i, Princeton Instruments), and detected by a silicon avalanche photodiode (id100–50, ID Quantique), which provides instrumental response function as 60 ps (FWHM).

## Electronic supplementary material

Supporting information
