# Supplementary material for: Reverse Anti-solvent Crystallization Process for the Facile Synthesis of Zinc Tetra(4-pyridyl)porphyrin Single Crystalline Cubes
Source: Sci Rep. 2017 May 31;7:2582. doi: 10.1038/s41598-017-02718-9 (PMC5451467; doi:10.1038/s41598-017-02718-9)
Supplement: Supplementary file 1 — Supporting information [file 41598_2017_2718_MOESM1_ESM.doc]

**Reverse Anti-solvent Crystallization Process for the Facile Synthesis of Zinc Tetra(4-pyridyl)porphyrin Single Crystalline Cubes**

. Yohwan Park,^1,2^ Misun Hong,^2,3^ Jin Young Koo,^1^ Minkyung Lee,^1,2^ Jinho Lee,^1,2^ Dae Jun Moon,^4^ So Hyeong Sohn,^2^ Taiha Joo,^2,*^ Woo Taik Lim,^4^,* Hyunseob Lim,^5^,* and Hee Cheul Choi^1,2^*

*^1^Center for Artificial Low Dimensional Electronic Systems (CALDES), Institute for Basic Science (IBS)*

*^2^Department of Chemistry, Pohang University of Science and Technology (POSTECH), 77 Cheongam-Ro, Pohang, Korea (790-784)*

*^3^Department of Chemistry, Korea Advanced Institute of Science and Technology, 291, Daehak-ro, Yuseong-gu, Daejeon 34141, Korea*

*^4^Department of Applied Chemistry, Andong National University, Andong 36729, Korea*

*^5^Center for Multidimensional Carbon Materials (CMCM), Institute of Basic Science (IBS)*

**Supporting Figures**


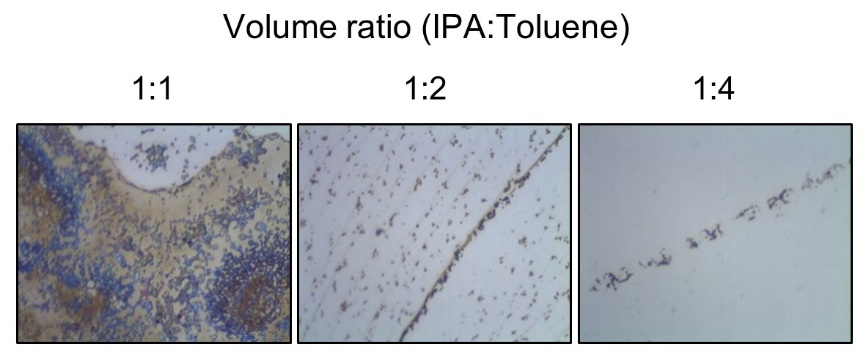
**Figure S1.** Optical microscopy images of drop casted ZnTPyP solution formed by c-ASC method which show no precipitates. The number above each figure represents the volume ratio of IPA to toluene. (The irregular blue solids in the microscope image are believed to be formed during the drop casting process)

To determine the accurate concentration of ZnTPyP/IPA and ZnTPyP/IPA/toluene solutions, the calibration curves have been determined by UV/VIS spectra from solutions of which concentrations are known as shown in Fig. S2.

**ZnTPyP/IPA**: Each absorption point shown in Fig. S2(b) was obtained from the solutions prepared by diluting solution with known concentration, then, the calibration curve was determined according to the following equation.

***A=39.4293c+0.8914*** *(A: intensity of absorption, c: concentration)*

**ZnTPyP/IPA/toluene**: Each absorption point shown in Fig. S2(d) was obtained from the solutions prepared by diluting solution with known concentration, then, the calibration curve was determined according to the following equation.

***A=311.5557c+0.1345*** *(A: intensity of absorption, c: concentration)*

**
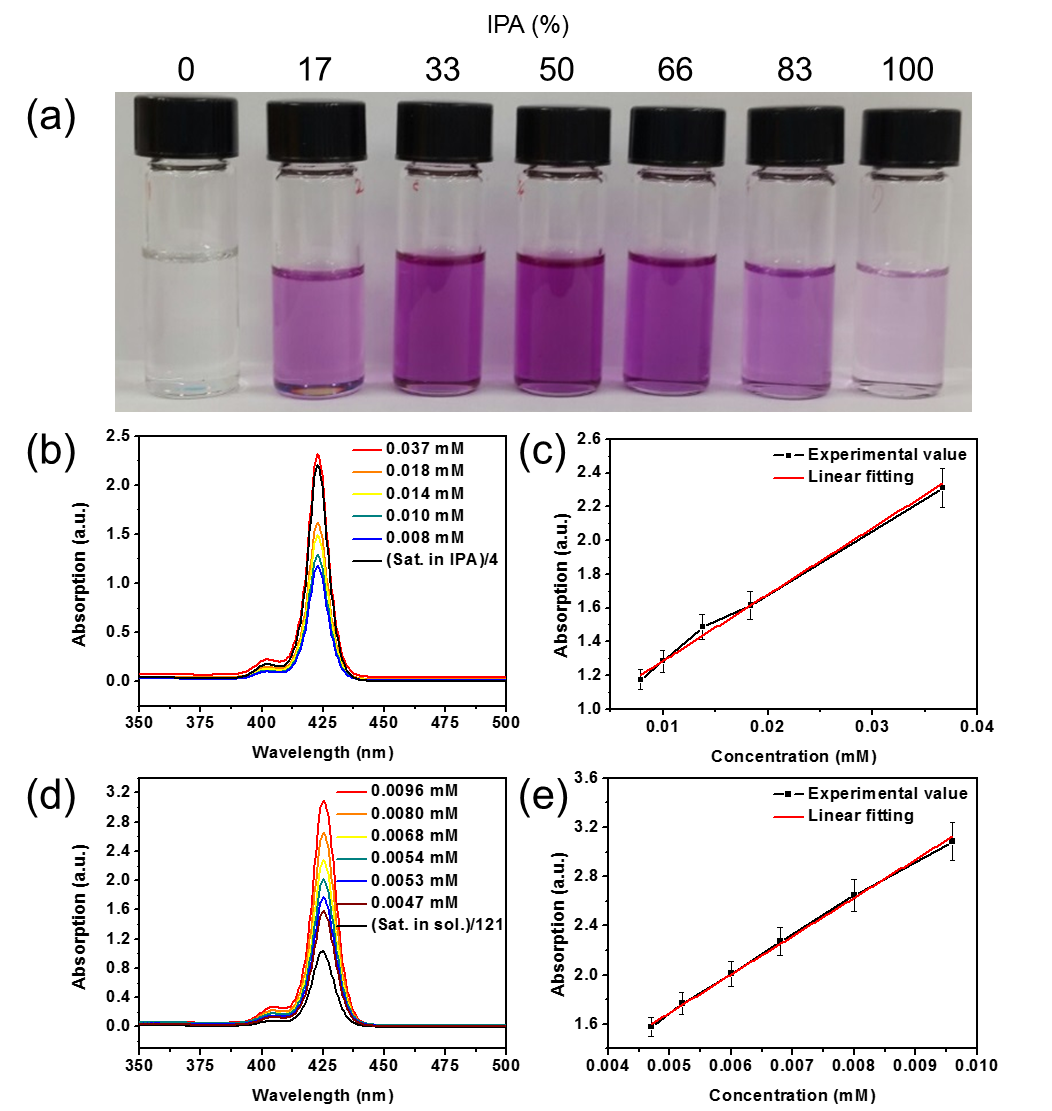
Figure S2.** (a) Optical photograph of ZnTPyP solutions depending on the IPA/toluene volume ratio after filtration. (b) UV-VIS spectra depending on the concentration of ZnTPyP dissolved in IPA. (c) Experimental values (black) and linear fitting (red) about absorption *vs.* concentration based on the results of (b). (d) UV-VIS spectra depending on the concentration of ZnTPyP dissolved in IPA/toluene solvent mixture. (e) Experimental values (black) and linear fitting (red) about absorption *vs.* concentration based on the results of (d).


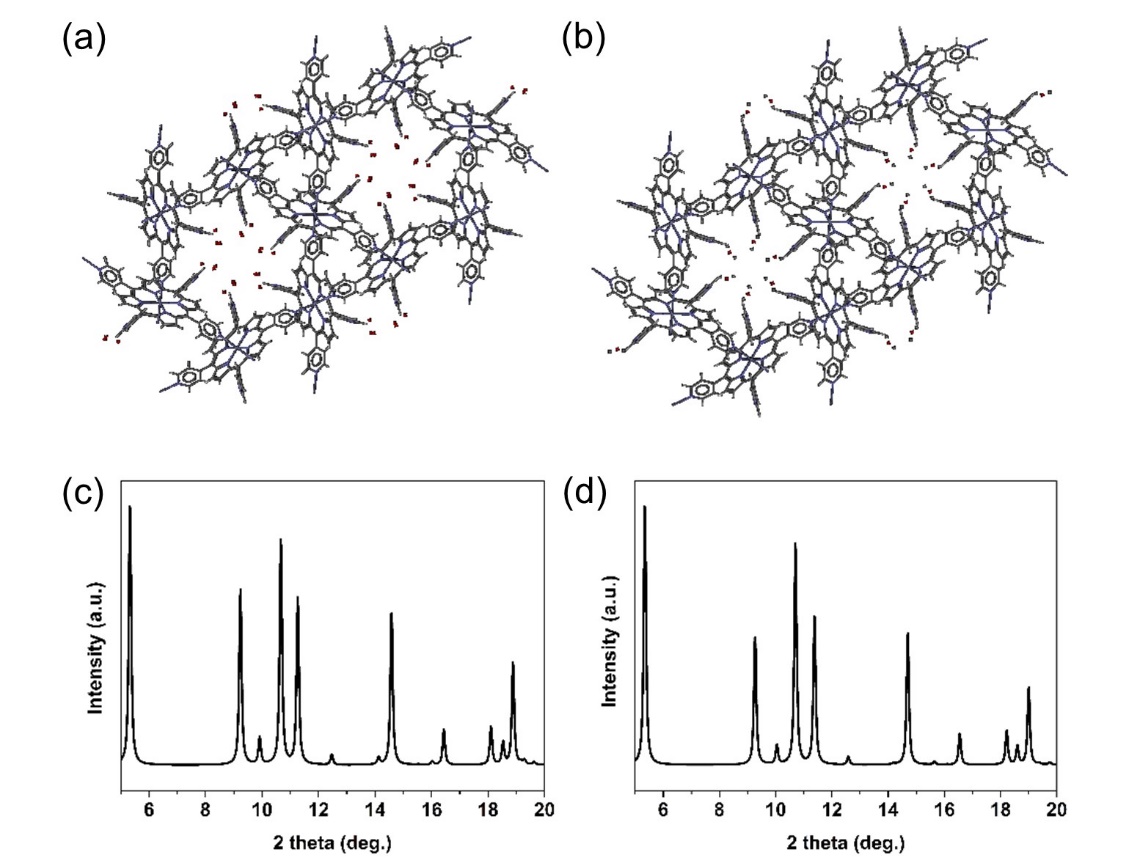
**Figure S3.** X-ray single crystal structure of ZnTPyP crystal space-filling representation along the c-axis direction of (a) H_2_O molecules solvated, and (b) MeOH and H_2_O molecules solvated crystal.^[1]^ (c and d) Powder XRD patterns of (a) and (b), respectively.


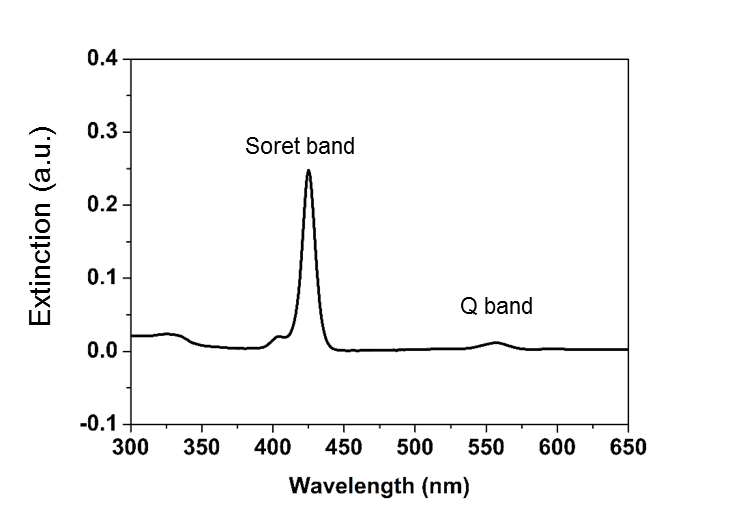


**Figure S4.** Absorption spectrum of ZnTPyP in binary solvent system, including toluene and IPA.


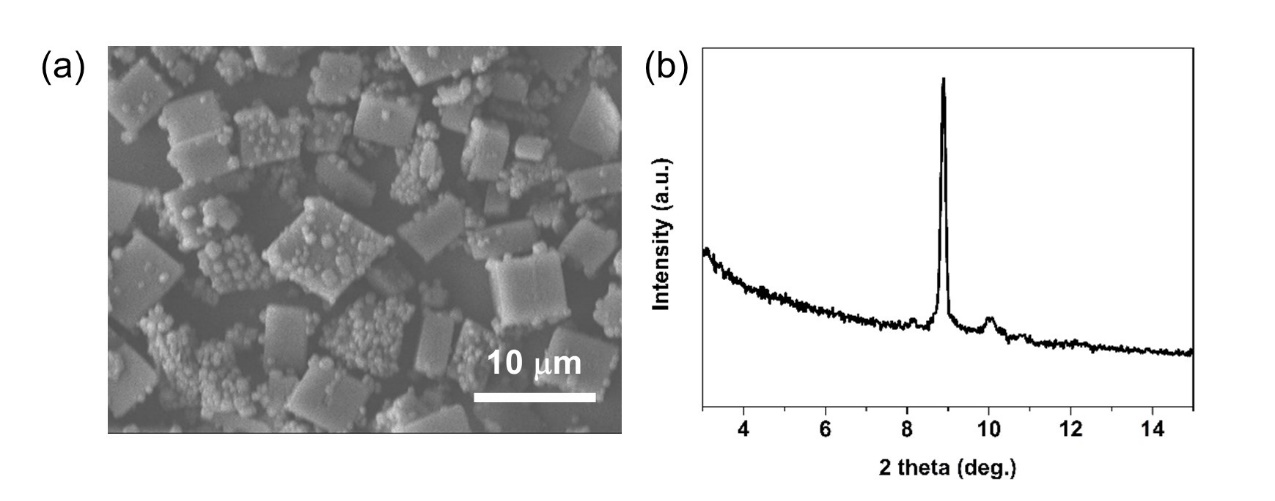
**Figure S5.** (a) SEM image and (b) XRD pattern grown by the *m*-ASC method with stirring for 3 h.


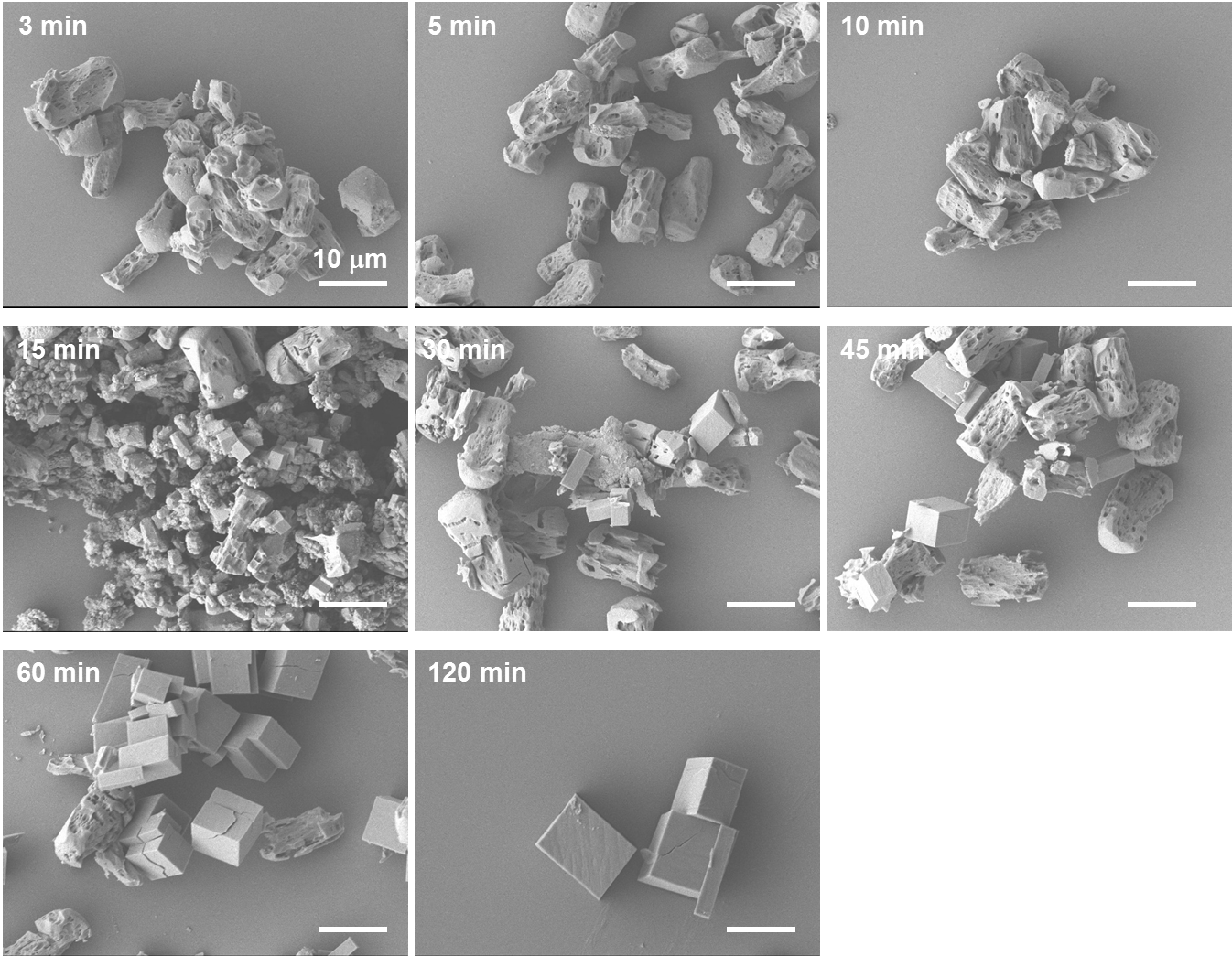


**Figure S6.** SEM images of crystal grown by the *r*-ASC method depending on time.


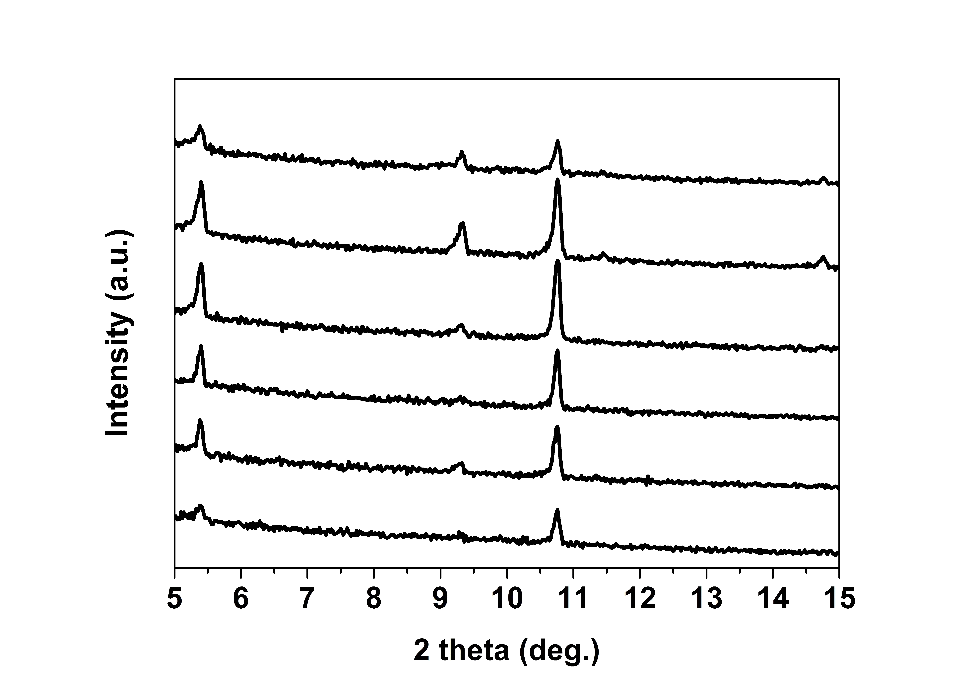


3 min

10 min

15 min

30 min

1 h

2 h

**Figure S7.** XRD pattern of crystal grown by the *m*-ASC method depending on time.

**
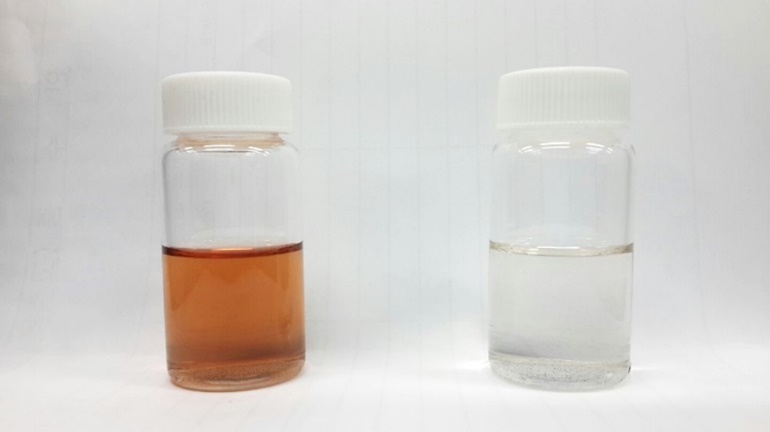
**

**Figure S8.** Optical photograph of C_70_ solution prepared by the *m*-ASC method (left) and the *r*-ASC method (right).


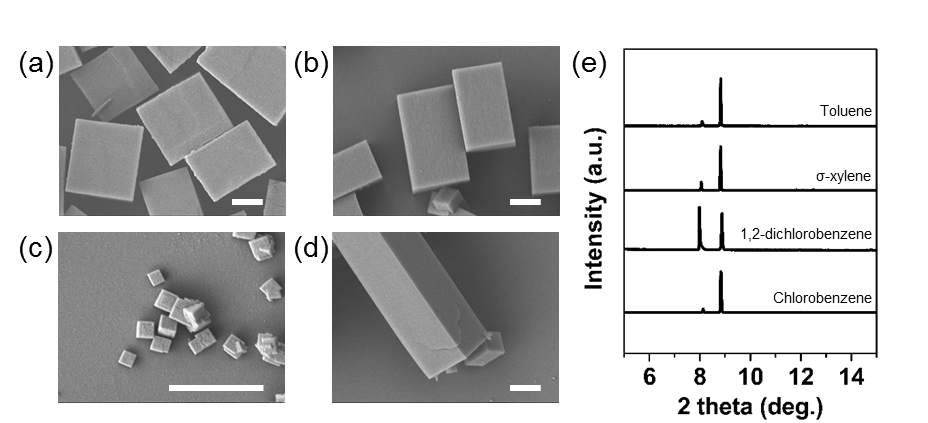


Figure S9. (a - d) SEM images of crystals grown by using toluene, *o*-xylene, chlorobenzene, and 1,2-dichlorobenzene, respectively, as anti-solvent by the *r*-ASC method (scale bar = 2 mm) and (e) XRD patterns corresponding to each crystal.

We also tested with other binary solvent systems, using chlorobenzene, 1,2-dichlorobenzene, and o-xylene as anti-solvents. Similar trends were confirmed in all cases, i.e., ZnTPyP cubes were precipitated by the *r*-ASC process, while the *m*-ASC process resulted in ZnTPyP powder regardless of the type of anti-solvent (fig. S8). Furthermore, other solute-solvent systems, e.g., C_70_ and mesitylene/IPA, have been attempted with the *r*-ASC process. In Fig. S7, while the color of the mixture solution using mesitylene as a first-added solvent is brown (left), the color of the solution using IPA as a first-added solvent following the *r*-ASC method is transparent (right). This result also implies that the first solvent role in forming the shell around C_70_ and IPA prevents C_70_ from dissolution by mesitylene. It is anomalous that different structures are precipitated depending on the order of the addition of solvent, although the final mixed solvents have identical concentrations of consisting solvents. These observations imply that a kinetic process occurs during the *r*-ASC, rather than the thermodynamic, process. Therefore, we proposed the FSSE mechanism of *r*-ASC based on the kinetic process between ZnTPyP and binary solvents.


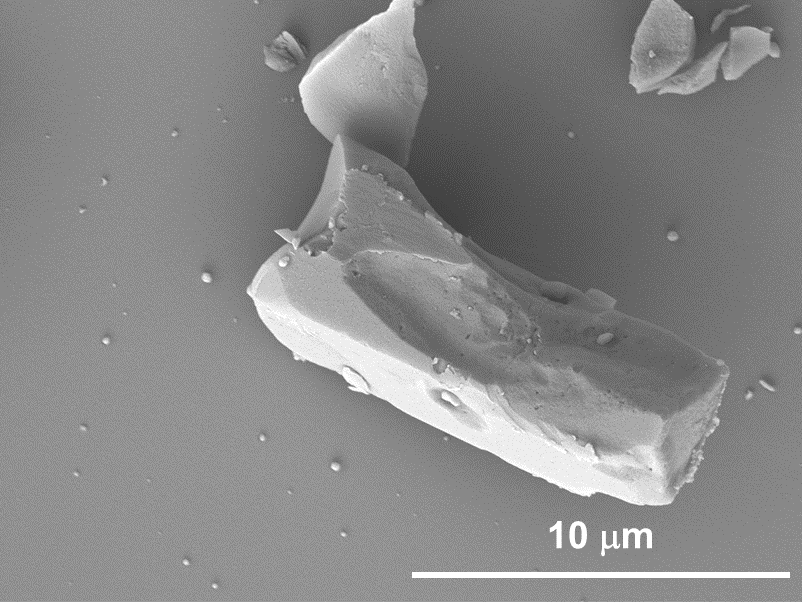


**Figure S10**. SEM image of initial ZnTPyP powder.


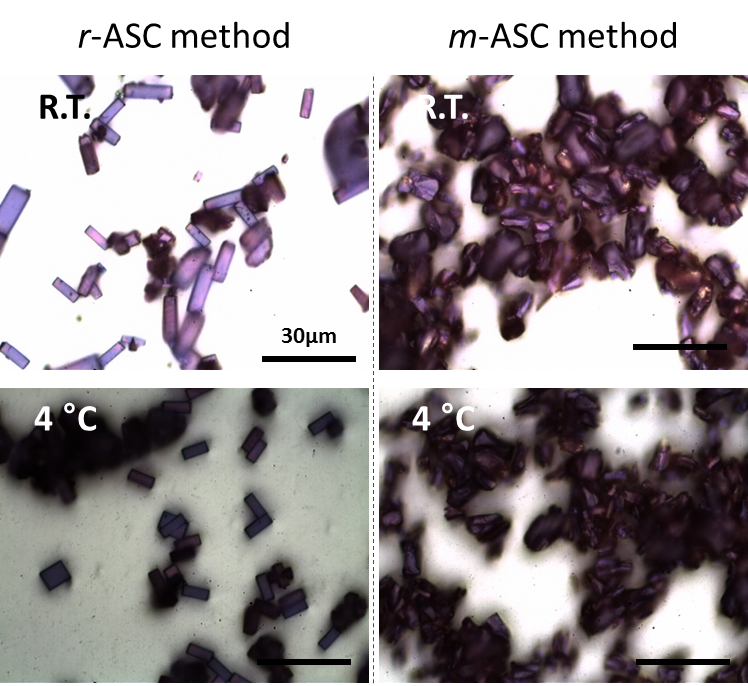


Figure S11. Optical images of crystals synthesized by *r*-ASC and *m*-ASC method depending on temperature.


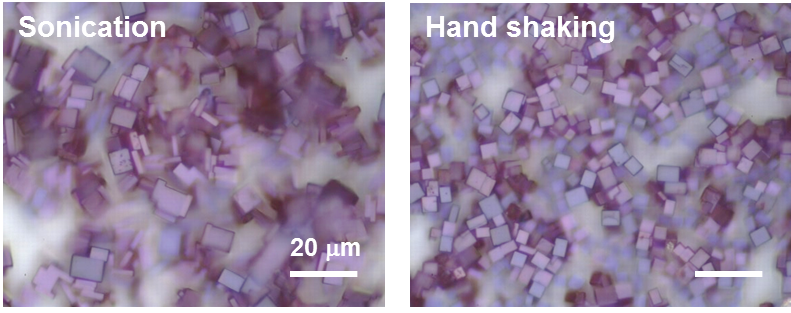


Figure S12. Optical microscopic images of crystals synthesized by r-ASC depending on mixing conditions.

**Reference**

S[1] H. Krupitsky, Z. Stein, I. Goldberg, C. E. Strouse*, J. Inclusion Phenom. Mol. Recognit. Chem***. 19**94*,* 18, 177-192.
